# Pertinence and development of cibd – clinical interview for bipolar disorder

**DOI:** 10.1192/j.eurpsy.2021.1646

**Published:** 2021-08-13

**Authors:** J. Azevedo, M. Martins, P. Castilho, C. Barreto, A.T. Pereira, A. Macedo

**Affiliations:** 1 Institute Of Psychological Medicine, Faculty of Medicine, University of Coimbra, Coimbra, Portugal; 2 Cineicc, Univerity of Coimbra, Coimbra, Portugal; 3 Psychology, University of Azores, Ponta Delgada, Portugal

**Keywords:** Assessment, CIBD, Clinical Interview, bipolar disorder

## Abstract

**Introduction:**

Bipolar disorder (BD) is frequently underdiagnosed and due to poor screening, the average time between onset of symptoms and diagnosis is more than 7-years (Mantere et al., 2004). Improper diagnosis has serious consequences in intervention (Ghaemi et al., 2001), and previous assessment instruments are now considered insufficient to detect intervention changes, and to provide a more functional and integrated view of BD.

**Objectives:**

Our study aims to develop a new DSM-5 based Clinical Interview for Bipolar Disorder (CIBD), providing criteria to diagnose BD, but also the individual’s perceptions dealing with BD symptoms. This interview follows the same structure of CIPD (Martins et al., 2015), which has shown acceptability by the participants and experts.

**Methods:**

CIBD was developed by a multidisciplinary team considering the DSM-5 criteria for Bipolar Disorders. There was a thorough research regarding assessment and evaluation of BD, and several suggestions from an international task force of specialist working with BD patients were considered, when writing the questions for the interview. A detailed description of CIBD development is presented. The authors of the interview have extended experience in the management and assessment of BD patients, and CIBD is now being assessed by a wider non-related panel, regarding pertinence and clarity.

**Results:**

Preliminary assessment and qualitative feedback from participants that were interviewed is shown, with an overall positive feedback.

**Conclusions:**

CIBD assesses both the diagnosis/presence of mood episodes (hypo/mania, and depressive) and symptoms’ psychosocial correlates. CIBD detects subtle changes caused by intervention adding a much needed recovery focused perspective.

**Disclosure:**

No significant relationships.

